# TGF-β induces growth suppression in multiple myeloma MM.1S cells via E2F1

**DOI:** 10.3892/ol.2020.12344

**Published:** 2020-12-02

**Authors:** Xialei Liu, Hui Guo, Yuting Wei, Chaonong Cai, Baimeng Zhang, Jian Li

Oncol Lett 14: 1884-1888, 2017; DOI: 10.3892/ol.2017.6360

Subsequently to the publication of this paper, an interested reader drew to the authors’ attention that the “Control” lane in the E2F1 panel of Fig. 2 appeared to be strikingly similar to the “24 h” TGF-β treatment lane in the E2F1 panel of Fig. 4.

Although the authors were unable to locate the original raw data to evaluate how the errors in figure compilation had occurred, they were able to repeat these experiments, and thereby could confirm that: i) E2F1 truly may can be down-regulated in the MM1s cell line through siRNA targeting E2F1; and ii) TGF-β can induce the immediate expression of E2F1 in the MM1s cell line within 24 h.

The revised versions of Figs. 2 and 4, containing the new data, are shown opposite. The authors regret the errors that were made in the compilation of the orginal figures, and are grateful to the editor of *Oncology Letters* for allowing them the opportunity to publish a Corrigendum. Furthermore, they apologise to the readership for any inconvenience caused.

## Figures and Tables

**Figure 2.**
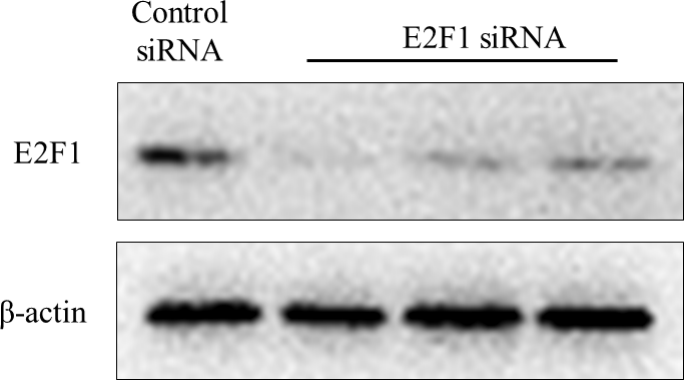
The efficiency of E2F1 knockdown by siRNA was verified by immunoblotting using an E2F1-specific antibody. Ctl, cells without transfection; siRNA, small interfering RNA; Ctl siRNA, cells transfected with unrelated siRNA controls; E2F1 siRNA, cells transfected with anti-E2F1 siRNA.

**Figure 4.**
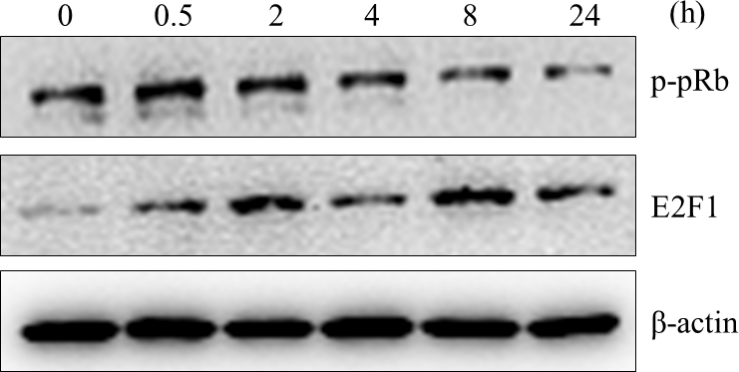
Western blot analysis of the phosphorylation state of pRb and total E2F1 protein levels in TGF-β-treated cells shows TGF-β induces E2F1 protein expression levels transiently. TGF-β, transforming growth factor-β; pRb, retinoblastoma tumor-suppressor protein.

